# Analysis and prediction of hand, foot and mouth disease incidence in China using Random Forest and XGBoost

**DOI:** 10.1371/journal.pone.0261629

**Published:** 2021-12-22

**Authors:** Delin Meng, Jun Xu, Jijun Zhao

**Affiliations:** 1 Complexity Science Institute, Qingdao University, Qingdao, Shandong, China; 2 State Key Laboratory of Resources and Environmental Information System, Institute of Geographic Sciences and Natural Resources Research, Chinese Academy of Sciences, Beijing, China; Amity University, INDIA

## Abstract

Hand, foot and mouth disease (HFMD) is an increasingly serious public health problem, and it has caused an outbreak in China every year since 2008. Predicting the incidence of HFMD and analyzing its influential factors are of great significance to its prevention. Now, machine learning has shown advantages in infectious disease models, but there are few studies on HFMD incidence based on machine learning that cover all the provinces in mainland China. In this study, we proposed two different machine learning algorithms, Random Forest and eXtreme Gradient Boosting (XGBoost), to perform our analysis and prediction. We first used Random Forest to examine the association between HFMD incidence and potential influential factors for 31 provinces in mainland China. Next, we established Random Forest and XGBoost prediction models using meteorological and social factors as the predictors. Finally, we applied our prediction models in four different regions of mainland China and evaluated the performance of them. Our results show that: 1) Meteorological factors and social factors jointly affect the incidence of HFMD in mainland China. Average temperature and population density are the two most significant influential factors; 2) Population flux has different delayed effect in affecting HFMD incidence in different regions. From a national perspective, the model using population flux data delayed for one month has better prediction performance; 3) The prediction capability of XGBoost model was better than that of Random Forest model from the overall perspective. XGBoost model is more suitable for predicting the incidence of HFMD in mainland China.

## Introduction

Hand, foot and mouth disease (HFMD) is a widespread infectious disease commonly caused by the enteric pathogen coxsackievirus A16 (Cox A16) and enterovirus 71 (EV71) [1, 2]. Since the first outbreak in 1957, it has gradually become a substantial burden throughout the Asia-Pacific region [1, 3]. China has reported an average of about two million cases of HFMD each year since 2008, making it the highest annual reported incidence compared with other childhood infectious diseases [1, 4]. Although the EV71 vaccination started since December 2015 [5], HFMD still causes millions of cases and hundreds of deaths every year [6]. It has become an increasingly serious public health problem.

Geographically, China covers a vast territory and has diverse climate zones, hence a more comprehensive analysis or prediction is needed for the entire country that covers varied meteorological conditions. Meanwhile, China has the largest population size and the most complex transportation network in the world. The population density varies greatly among provinces and the population flux has a strong seasonality. These make China a good, but challenging candidate for the study and prediction of HFMD incidence. HFMD incidence has different seasonal patterns in China. In temperate climatic zone HFMD incidence time series has an annual peak in summer, while in tropical and subtropical climatic zones, incidence may have multiple peaks every year [1, 2]. Predicting the incidence of HFMD and analyzing its influential factors are of great significance to its prevention. Different kinds of models have been developed to predict the incidence of infectious diseases. The autoregressive integrated moving average (ARIMA) model is a commonly used time series prediction model [7–9]. Time series model has a good predictive capability, but it does not consider the change in patterns of potential influential factors. The correlations between HFMD incidence and influential factors can be analyzed using linear regression model [10]. To capture the nonlinear characteristics of the predictor variables, some traditional nonlinear models are used, and the generalized additive model (GAM) is one of them. But, statistical parameters such as mean, standard deviation, and correlation are highly sensitive to outliers. Therefore, traditional statistical procedures may severely affect the performance of the final model. With the booming development of artificial intelligence, machine learning has shown its advantages in analysis and prediction. In this study, we used machine learning methods to predict HFMD incidence. Machine learning methods can not only capture the nonlinear relationship between different factors, but also achieve better performance for non-linear time series. We perform our analysis and prediction by using two different machine learning algorithms, Random Forest and eXtreme Gradient Boosting (XGBoost). These two methods are applied in many fields, such as the classification and regression of gene sequences and the monitoring and tracking of human motion [11, 12].

In this paper, we aim to examine the association between HFMD incidence and potential influential factors for 31 provinces in mainland China, and to establish Random Forest and XGBoost prediction models on HFMD incidence. In order to compare the performance of our prediction models in different regions, we also perform cluster analysis on meteorological factors for 31 provinces using the K-means algorithm. A comparison between Random Forest model and XGBoost model is conducted using evaluation criteria to determine which was more suitable for predicting the HFMD incidence in specific region. In addition, we also studied the delayed effect of population flux on HFMD incidence in mainland China. Some studies have found the delayed effect of population mobility on transmission rate of different diseases, such as rubella, HFMD, and COVID-19 [3, 13, 14]. Unlike meteorological factors, population flux affect HFMD incidence because people bring viruses from one place to another and this process takes much longer time than the direct effect from meteorological factors. The incubation period of HFMD is three to five days, and the infectious period is seven to 10 days [15]. Hence, an infected individual who travels from one place to another may take up to two weeks to infect a susceptible individual.

Our study involves 31 provinces, municipalities and autonomous regions in mainland China. They are all called “provinces” in this paper. Our study does not include Hong Kong SAR, Macau SAR and Taiwan.

## Methods

### Selection of prediction factors

In this study, we consider five meteorological factors and two social factors, as potential influential factors of HFMD incidence.

Meteorological factors affect the survival rate of viruses or affect human behavior that may further affect the transmission and incidence of HFMD. Some biological laboratory studies have shown that the stability of enteroviruses is affected by meteorological factors such as temperature and relative humidity [16]. A number of studies have found that HFMD incidence is commonly related to meteorological factors such as average temperature and relative humidity [16–18]. However, findings of different studies about influential factors are not consistent with each other. For example, a study that provided quantitative evidence showed that as relative humidity increases, the number of HFMD cases also increased significantly [18]. Another study pointed out that no correlation between relative humidity and HFMD was observed [19]. Such inconsistencies also exist in the relationship of other meteorological factors and HFMD cases. We chose temperature and relative humidity as predictors, in order to further explore the effects of them on HFMD incidence.

On the other hand, climate will greatly affect human social behavior, thereby affecting the transmission of some human infectious diseases. A study illustrated that coxsackieviruses can remain viable on hard, nonporous surfaces for two weeks in conditions of high temperature and low humidity [20]. The longer the virus survives in the environment, the more amount of the virus can be accumulated in the environment and cause more transmission of the virus [21]. It means that the contact rate of people also plays an important role in the transmission of HFMD. For example, compared to cold and windy winters, children are more likely to go outdoors to the playground in summer. Direct contact with contaminated toys and surfaces may also cause the spread of the virus [16]. Therefore, some meteorological factors that affect people’s social behavior, such as precipitation, wind speed, and sunshine hours, are also selected as predictors in this study.

In another stream of studies focusing on modeling transmission rate of diseases rather than studying incidence directly, it is found that some social factors, including population flux and population density, are potential factors affecting disease transmission. Population flux caused by raining season or simply due to social behavior has effect in the transmission of measles or rubella [13, 22]; school terms can increase transmission rate in measles and other childhood infectious diseases [23]. Seasonal population flux in China was seen as a piece of puzzle, which is lacking in studies of HFMD. Population density is also an influential factor that cannot be ignored [24]. This stream of studies points out the complex nature of disease transmission that cannot be solely described by meteorological factors. Similarly, when we predict HFMD incidence, we should not only focus on the relationship between meteorological factors and HFMD incidence, but also consider social factors including population flux and population density.

### Dataset collection and processing

There are five datasets utilized in our study. The details of datasets are shown in Table 1. Incidence dataset consists of monthly cases and incidence reports of HFMD for 31 provinces from January 2009 to December 2017. Meteorological dataset are monthly meteorological data for 31 provinces from January 2009 to December 2017, including average precipitation, average wind speed, average temperature, average relative humidity, and sunshine hours. Population flux dataset is the daily population flux, including inflow and outflow, of all 31 provinces in the year 2017. Passenger traffic dataset is the annual passenger traffic data from 2009 to 2017. Demography dataset contains population data at the year-end from 2009 to 2017, and it is used to calculate the population density of 31 provinces.

**Table 1:** Datasets in this study.

Datasets	Names	Duration	Temporal resolution	Spatial resolution	Sources
I	Incidence dataset	2009–2017	Month	31 provinces	The Data-Center of China Public Health Science [6]
II	Meteorological dataset	2009–2017	Month	31 provinces	China Meteorological Data Service Centre [25]
III	Population flux dataset	2017	Day	31 provinces	Tencent Location Big Data [26]
IV	Passenger traffic dataset	2009–2017	Year	31 provinces	The 2018 China Statistical Yearbook [27]
V	Demography dataset	2009–2017	Year	31 provinces	The 2018 China Statistical Yearbook [27]

**Fig 1:**
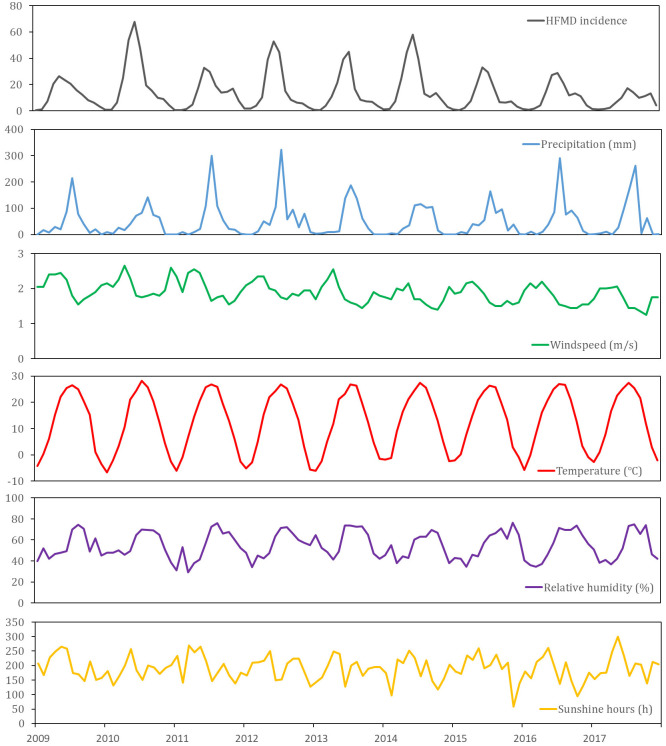
Time series of HFMD incidence and meteorological factors in Beijing from January 2009 to December 2017. Time series is available for every province in our datasets.

We first filled in the missing values in meteorological dataset. Since the missing values only exists in a few months of the year, we used the average of the data values in other years to estimate the missing values. For example, the sunshine hours in Beijing in May 2016 is a missing value. It can be estimated as the average of the real values of May from 2009 to 2017. Taking Beijing as an example, Fig 1 shows an excerpt of incidence dataset and meteorological dataset.

Next, we combined the daily data in population flux dataset into monthly data so that it can match the temporal resolution of meteorological dataset. Our initial analysis of population flux dataset found that the population flux had an annual seasonal pattern. We also found a long term increasing trend of passenger traffic dataset. For population flux dataset, we combined its annual seasonal pattern with the long term increasing trend extracted from passenger traffic dataset by using a linear regression model. So that population flux dataset can match the duration of meteorological dataset. We used R 4.0.5 to perform linear regression model. After that, we subtracted the monthly population outflow from the monthly population inflow as net population inflow, and named this net population inflow as population flux data for January 2009 to December 2017.

The population data of each province in demography dataset was divided by the area of each province to obtain the population density of 31 provinces from 2009 to 2017. We assumed that the population density of each province does not change within a year, so we regarded annual population density data as monthly population density data for 12 months of that year, enabling population density data to match the temporal resolution of the meteorological dataset.

In descending order according to the number of provinces, the datasets we utilized in this study include the national dataset, the regional dataset, and the provincial dataset. Monthly HFMD incidence, normalized monthly meteorological dataset, normalized monthly population flux data, and normalized monthly population density data were used as input data for our prediction models. Among our input data, HFMD incidence is the dependent variable, five meteorological factors and two social factors are the predictors. We carried out data normalization on all predictors using min-max normalization methods. Whether the original data is positive or negative, the range of all data values will be between 0 and 1 after processing. This work makes all predictors have the same weight and avoids the complexity of data analysis. We used R 4.0.5 to perform min-max normalization.

### Random Forest and XGBoost

In this study, we used Random Forest to examine the association between HFMD incidence and potential influential factors in mainland China. We used Random Forest and XGBoost to establish prediction models on HFMD incidence in mainland China.

**Fig 2:**
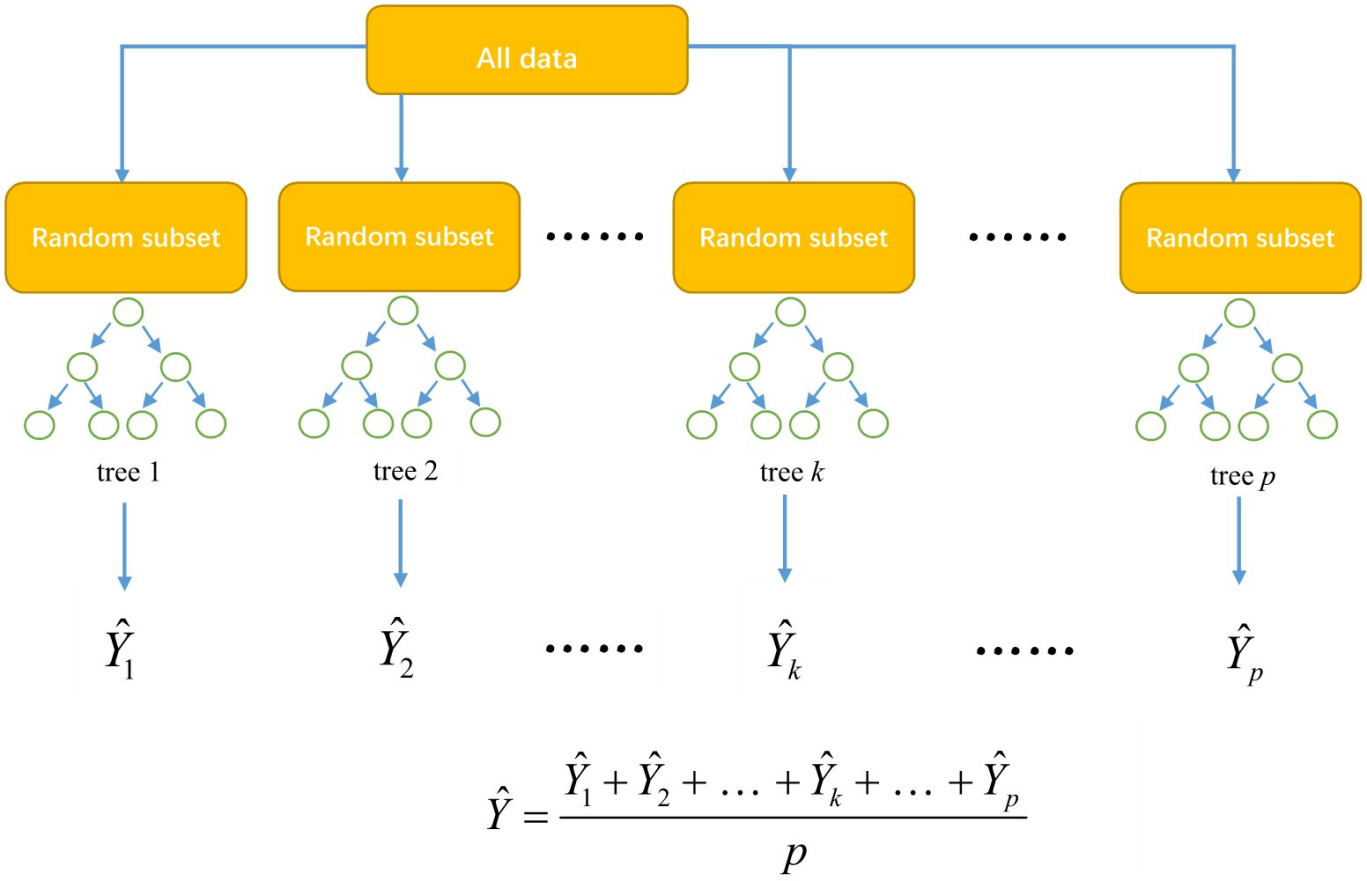
Framework of Random Forest algorithm.

The random selection of data and the random selection of features are the characteristic of Random Forest algorithm, proposed by Leo Breiman [28]. In our study, the features are predictors such as average temperature, average humidity, population flux, etc. The framework of Random Forest algorithm is shown in Fig 2. Random Forest uses bootstrap aggregation ensemble method to randomly construct *p* random sample subsets. In the predicting process, decision tree *k* (*k* = 1, 2, …, *p*) has an output *Ŷ*_*k*_. Then the average result *Ŷ* of *p* decision trees is the final result of Random Forest. The final model estimates the importance of each predictor by checking how much the prediction error has increased.

XGBoost algorithm was first proposed by Chen Tianqi and Carlos Guestrin in 2016 [29]. Compared with Random Forest, XGBoost uses a gradient boosting method, in which the model-building process is carried out in stages. In this algorithm, each tree is generated to reduce the value of the objective function, thereby improving the regression result of the model. In each iteration of this algorithm, a function *f*_*t*_ is added to minimize the following objective function:

$$Obj^{\left(t\right)}=\sum_{q=1}^{m}l(z_{q},{\hat{z}}_{q}{}^{\left(t-1\right)}+f_{t})+\Omega (f_{t})+C$$ (1)

where *m* is the numbers of input variables, *z*_*q*_ is the observed value of the variable *q*, *ẑ*_*q*_^(*t*-1)^ is the predicted value of the variable *q* at iteration *t*-1, and *l* is a differentiable convex loss function that measures the difference between the observed value *z*_*q*_ and the prediction result. In iteration *t*, with the addition of function *f*_*t*_, the prediction result is equal to the sum of *ẑ*_*q*_^(*t*-1)^ and *f*_*t*_. Ω is the regularization term that punishes the complexity of *f*_*t*_, and *C* is the constant generated during the iteration [29].

With *%IncMSE* as the evaluation standard, we obtained the evaluation of the importance of different influential factors. *%IncMSE* is calculated as the average difference of the variables’ mean square error from the original dataset and sets of randomly arranged variables [30]. The importance of influential factors is proportional to *%IncMSE* value. We used the randomForest package in R 4.0.5 to perform Random Forest analysis. The XGBoost algorithm is executed with the sklearn package in Python 3.7.0.

### Cluster analysis

In order to compare the performance of our prediction models in different regions, we used cluster analysis to classify provinces with similar climatic conditions into one region, and then evaluated our prediction models in each region.

We utilized the K-means algorithm to cluster 31 provinces in mainland China [31]. We extracted the average, maximum, and minimum values of five meteorological factors in 108 months from meteorological dataset and combined these 15 meteorological items into a cluster-specific dataset as the input data for our cluster analysis. The dimension of the cluster-specific dataset is 31 × 15. We used the factoextra package in R 4.0.5 to perform K-means algorithm.

**Fig 3:**
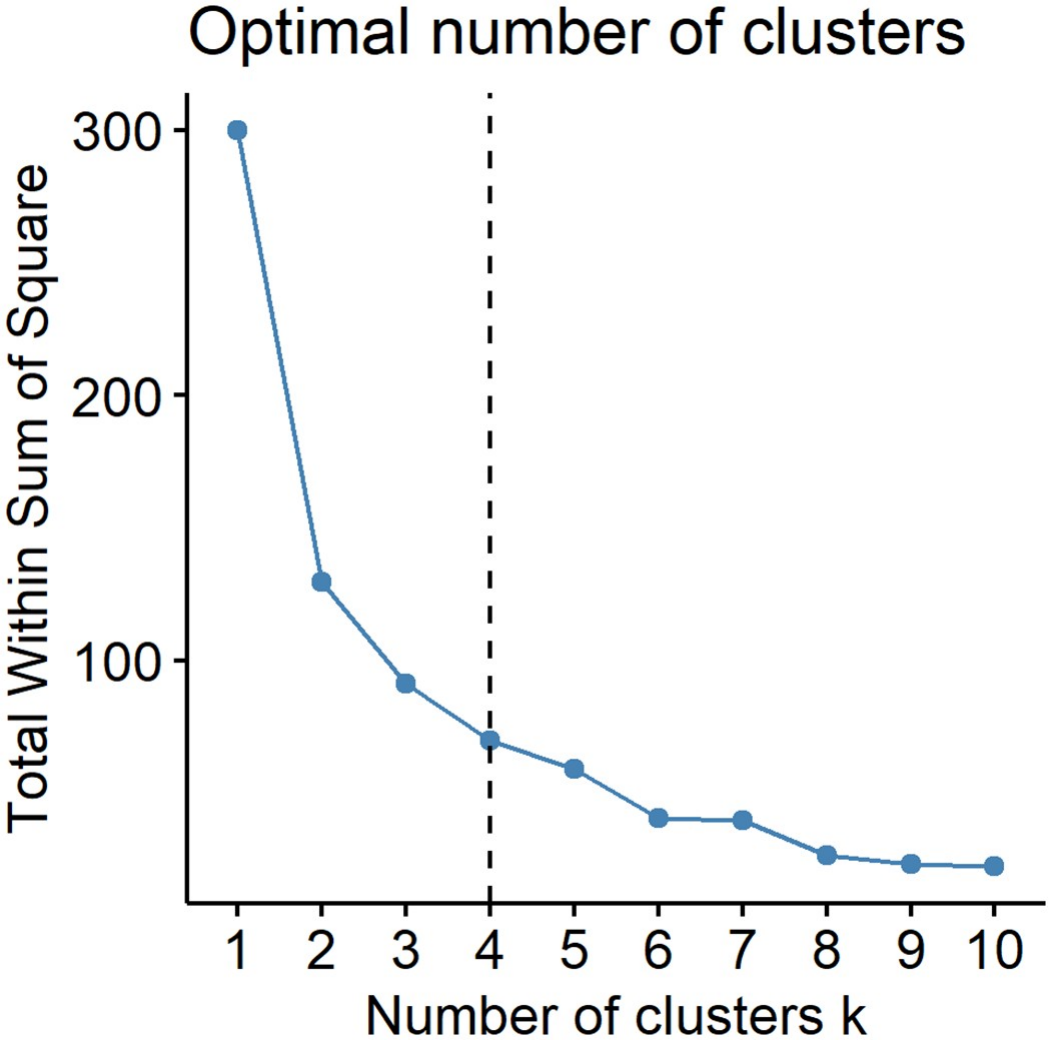
Determination of the optimal number of clusters.

**Fig 4:**
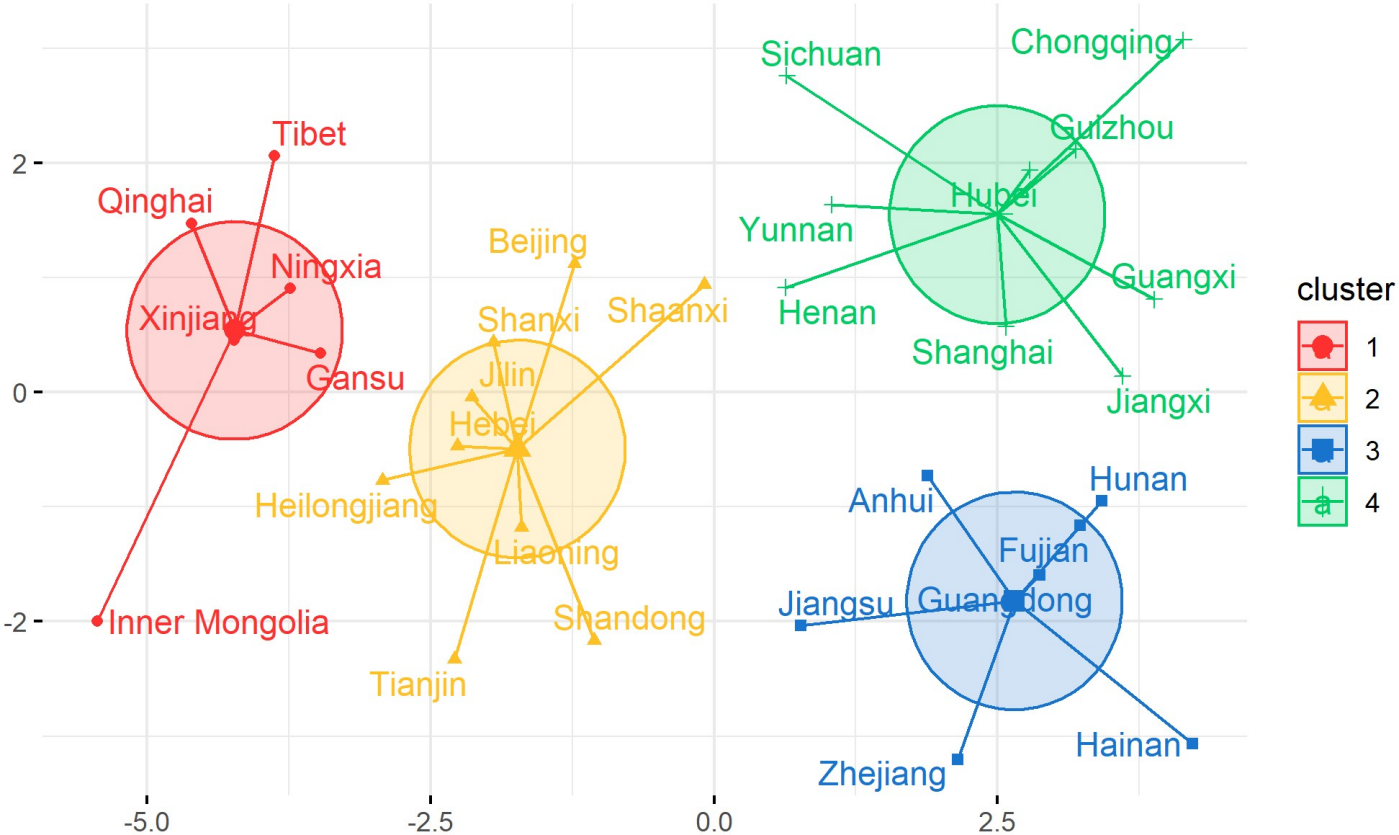
Clusters for 31 provinces in mainland China, through color coding.

The Elbow method was applied to find the optimal number of clusters [32]. It uses total within-cluster sum of square (WSS) as the evaluation standard, which measures the compactness of the clustering. The optimal number of clusters k is 4 (Fig 3). So we grouped 31 provinces in mainland China into four clusters (Fig 4).

### Evaluation criteria for model comparison

In this study, comparison of prediction models comes from the following two aspects. One is to compare the impact of population flux with different time delay on the prediction capability of models. The other is to compare the performance of two prediction models.

The purpose of the first comparison is to find out whether there is any delayed effect of population flux on HFMD incidence. For this purpose, we reconstructed our input dataset with population flux data with a one-month and two-month delay, respectively. We lined up the five meteorological factors, population density data and population flux data as columns, then shifted the population flux column by removing its first one or two data and adding one or two extrapolated data at the end of column for one-month or two-month delay respectively.

We used the mean squared error (MSE) and the explained variance score (EVS) together as the evaluation criteria. If *ŝ*_*α*_ is the predicted value of the *α*-th sample, and *s*_*α*_ is the corresponding observed value, then the mean squared error estimated over *n* samples is defined as:

$$MSE=\frac{1}{n}\sum_{\alpha =1}^{n}{\left(s_{\alpha }-{\hat{s}}_{\alpha }\right)}^{2}$$ (2)

If *ŝ* is the set of all predicted values, and *s* is the set of all corresponding observed values, and *Var* is variance, the square of the standard deviation, then the EVS is estimated as:

$$EVS=1-\frac{Var\{s-\hat{s}\}}{Var\{s\}}$$ (3)

The value range of the EVS is [0,1]. The best possible score is 1, lower values are worse. A score of 1 is not realistically attainable, but a score closer to 1 is deemed to have better prediction performance. It is worth noting that the comparison of models and the comparison of influential factors have different evaluation criteria.

## Results

### Ranking of factors affecting HFMD incidence

We analyzed the impact of potential influential factors on HFMD incidence using Random Forest model, and then ranked the influential factors according to *%IncMSE*. We used the national dataset and the provincial dataset in this section. In the analysis of each province, population density data was not taken into account because the difference between population density data within nine years in the same province is negligible.

Table 2 presented the *%IncMSE* values and the ranking of different influential factors based on the national dataset. We found that meteorological factors and social factors jointly affect the incidence of HFMD in mainland China. Among these influential factors, average temperature has the greatest impact on HFMD incidence. The importance of population density and population flux on HFMD incidence ranked second and third respectively.

**Table 2:** Ranking of *%IncMSE* values for influential factors based on the national dataset.

Factors	*%IncMSE*
Temperature	45.195
Population density	34.252
Population flux	26.533
Wind speed	24.791
Sunshine hours	24.398
Relative humidity	19.798
Precipitation	17.726

Table 3 presented the ranking of different influential factors based on the provincial dataset. According to Table 3, the impact of influential factors on HFMD incidence has different performance in different provinces. Average temperature is the most important factor affecting HFMD incidence in 24 provinces, accounting for 78%. Meanwhile, 16% of provinces regard population flux as the most important factor. In addition, average temperature and population flux are ranked as the top two important factors among 94% and 58% of all provinces.

**Table 3:** Ranking of influential factors affecting HFMD incidence in 31 provinces of mainland China.

	Temperature	Population flux	Precipitation	Relative humidity	Wind speed	Sunshine hours
Beijing	1	2	3	4	5	6
Jiangsu	1	2	3	4	5	6
Anhui	1	2	3	4	5	6
Shaanxi	1	2	3	4	5	6
Qinghai	1	2	3	4	5	6
Zhejiang	1	2	3	4	6	5
Yunnan	1	2	3	5	4	6
Gansu	1	2	3	5	4	6
Ningxia	1	2	3	5	4	6
Shandong	1	2	3	5	6	4
Inner Mongolia	1	2	3	6	4	5
Tianjin	1	2	3	6	5	4
Jiangxi	1	3	2	4	6	5
Jilin	1	4	2	3	5	6
Shanxi	1	4	3	2	5	6
Fujian	1	4	5	2	6	3
Hunan	1	5	2	3	6	4
Guangdong	1	5	2	3	6	4
Liaoning	1	5	3	2	4	6
Hainan	1	5	4	3	6	2
Heilongjiang	1	6	2	3	5	4
Guangxi	1	6	2	3	5	4
Hebei	1	6	2	4	3	5
Henan	1	6	2	5	3	4
Hubei	2	1	3	5	6	4
Guizhou	2	1	3	6	5	4
Xinjiang	2	1	4	6	5	3
Shanghai	2	1	5	3	6	4
Tibet	2	3	5	1	4	6
Chongqing	4	1	5	2	3	6
Sichuan	4	2	5	3	1	6
Percentage of 1	0.78 (24/31)	0.16 (5/31)	0	0.03 (1/31)	0.03 (1/31)	0
Percentage of 2	0.16 (5/31)	0.42 (13/31)	0.26 (8/31)	0.13 (4/31)	0	0.03 (1/31)

The smaller the number, the greater the influence of this factor on HFMD incidence. 1 represents the greatest influence on HFMD incidence, and 6 represents the least.

### Prediction models

Based on the national dataset, we established our prediction models on HFMD incidence using Random Forest and XGBoost algorithms respectively. The input data from January 2009 to December 2017 were randomly divided into two parts: a training set (86 months) and a test set (22 months). Hence, a total of 3,348 sets of influential factors with HFMD incidence ranged from 0 to 167.12 are divided into 2,678 training samples and 670 testing samples.

**Fig 5:**
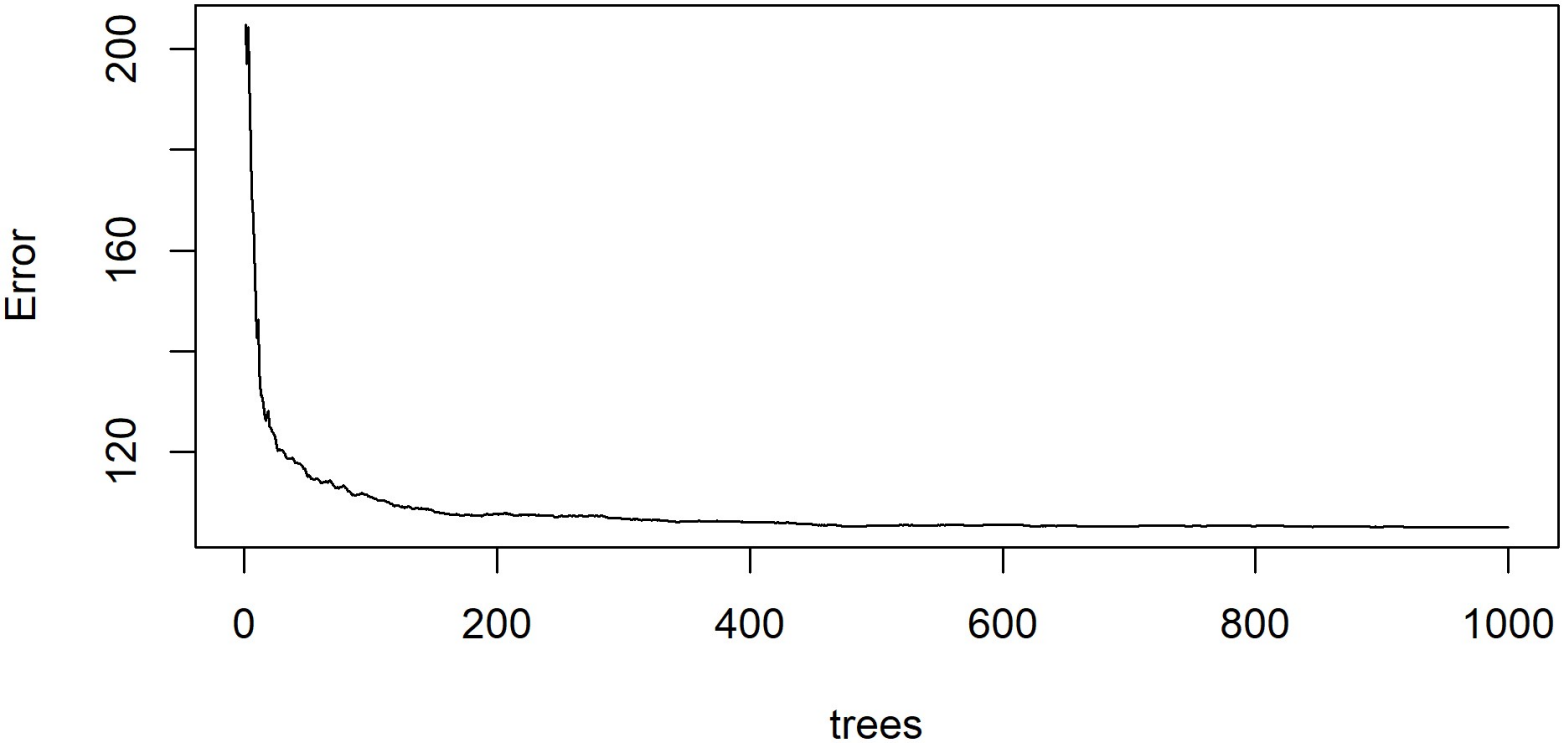
The error of Random Forest model against the number of decision trees.

The Random Forest model in R has two critical parameters, ntree and mtry, which are the number of decision trees and the number of features required to construct each tree. We plotted the relationship between Random Forest model prediction error and the number of decision trees to find out the suitable number of decision trees. As shown in Fig 5, the model error decreases as the number of trees increases. When the number of trees reaches 400, the model error is basically stable. But when the number of trees is between 400 and 500, the error of the model still fluctuates slightly. Therefore, we choose 500 as the number of decision tree for our Random Forest model, as well as XGBoost model.

By repeatedly adjusting the parameter mtry, Random Forest model with the best performance is obtained: ntree = 500, mtry = 2. We used GridSearchCV method in scikit-learn to implement parameters tuning. The XGBoost model with the best performance is obtained: n_estimators = 500, eta = 0.08, gamma = 0.1, max_depth = 5, min_child_weight = 4.

**Fig 6:**
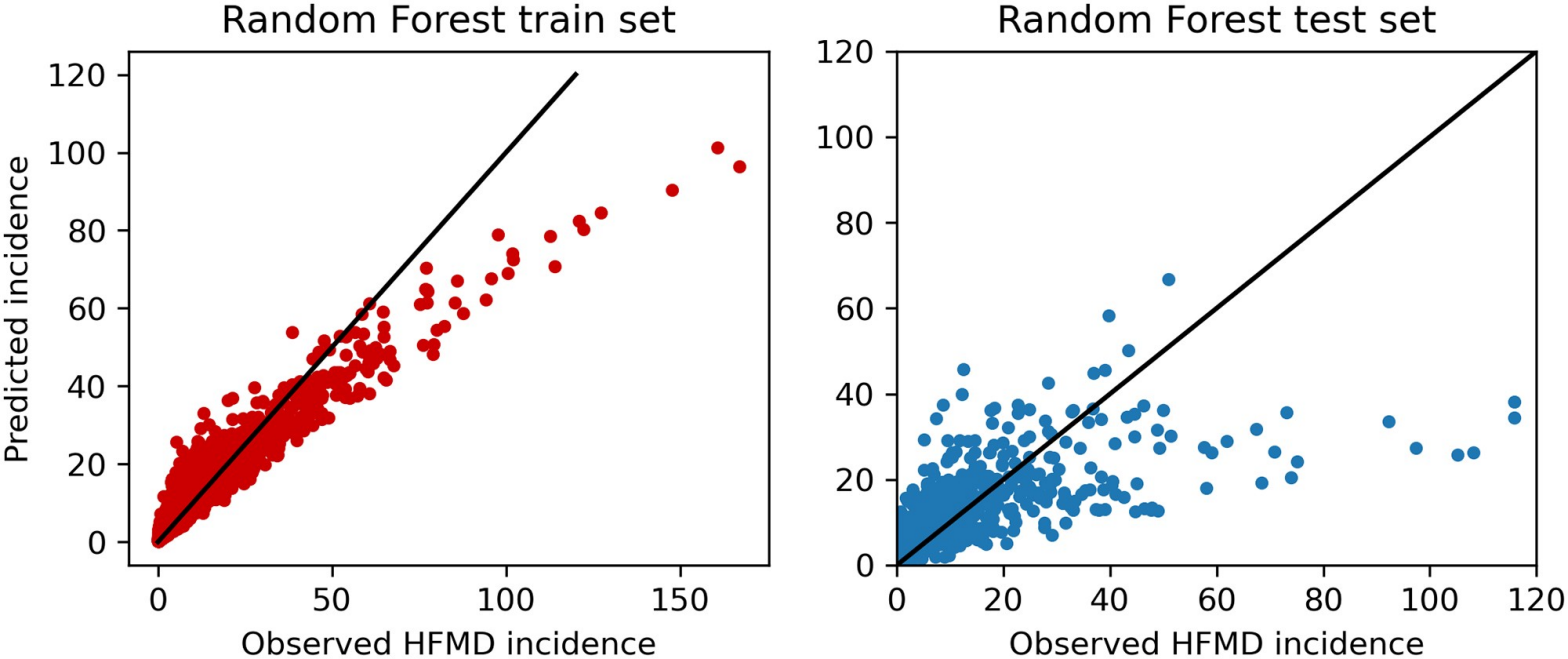
Predicted incidence against observed HFMD incidence using Random Forest model.

**Fig 7:**
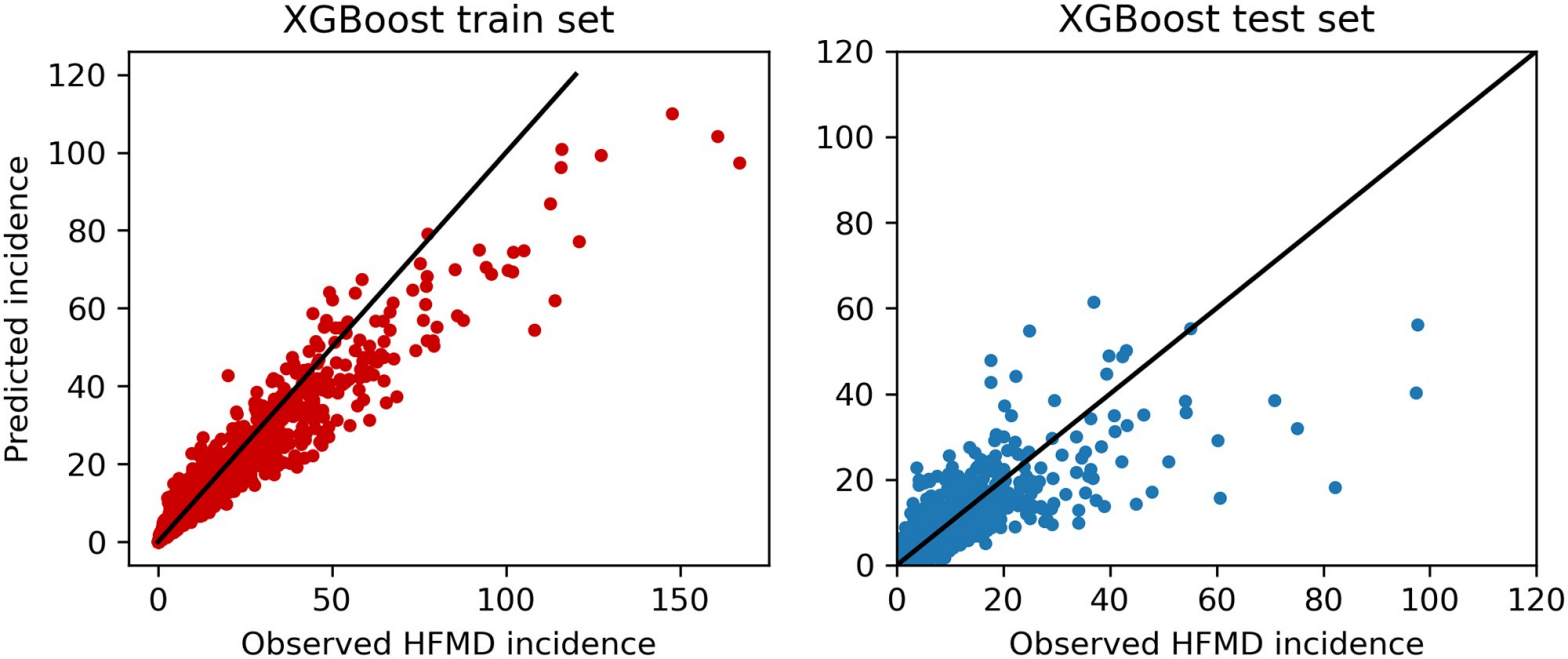
Predicted incidence against observed HFMD incidence using XGBoost model.

Figs 6 and 7 are scatter plots of predicted incidence against observed HFMD incidence for our train set and test set using different prediction models. Each point (*x*, *y*) represents the pair of one observation and one prediction. If the point is near the line *y = x*, the predicted value is consistent with the historically expected in that month.

### Model comparison

We used the MSE and the EVS together as the evaluation criteria to quantify the performance of the two models (Random Forest and XGBoost) and rank them in terms of performance. Meanwhile, the most suitable delayed time (no delay, or one-month delay, or two-month delay) of population flux can be find out in predicting HFMD incidence in different regions of mainland China. We used the national dataset and the regional dataset in this section. We also put our clustering results in Table 4 for viewing purposes.

**Table 4:** Regional division for 31 provinces in mainland China.

Clusters	Provinces
1	Tibet, Qinghai, Ningxia, Xinjiang, Gansu, Inner Mongolia
2	Beijing, Shaanxi, Shanxi, Jilin, Hebei, Heilongjiang, Liaoning, Tianjin, Shandong
3	Anhui, Hunan, Fujian, Guangdong, Jiangsu, Zhejiang, Hainan
4	Sichuan, Chongqing, Guizhou, Hubei, Yunnan, Henan, Shanghai, Guangxi, Jiangxi

The results of our model comparison are presented in Table 5. Except for Cluster 3, XGBoost prediction model has a smaller MSE and a larger EVS than Random Forest model in all clusters. Our XGBoost model shows better prediction performance for predicting the incidence of HFMD in mainland China.

**Table 5:** MSE and EVS in different regions with different time delay using Random Forest and XGBoost.

		MSE			EVS		
		No delay	One-month delay	Two-month delay	No delay	One-month delay	Two-month delay
Country level	Random Forest	103.51	104.41	104.14	0.5429	0.5390	0.5403
	XGBoost	74.88	**70.69**	87.72	0.5593	**0.5613**	0.5572
Cluster 1	Random Forest	13.75	14.80	15.08	0.5991	0.5688	0.5612
	XGBoost	6.74	**5.43**	10.62	0.6422	**0.7386**	0.6269
Cluster 2	Random Forest	36.99	38.82	38.71	0.6534	0.6364	0.6375
	XGBoost	**28.98**	39.17	50.36	**0.6991**	0.6627	0.5982
Cluster 3	Random Forest	208.94	203.51	206.42	0.4385	**0.4531**	0.4453
	XGBoost	242.31	191.78	**171.29**	0.3927	0.3905	0.4350
Cluster 4	Random Forest	130.10	134.81	133.95	0.4924	0.4744	0.4780
	XGBoost	76.29	**70.94**	91.23	0.5708	**0.6338**	0.5981

The best evaluation criteria value for each region have been marked in **bold**.

As shown in Table 5, our results have strong regional characteristics. Population flux has different delayed effect in affecting HFMD incidence in different regions based on XGBoost model, but such delayed effect is difficult to capture by Random Forest model. XGBoost model with one-month delayed population flux has the best performance in prediction HFMD incidence at the country level. The same conclusion can be drawn from Cluster 1 and Cluster 4, in which most provinces are located in the western inland region of China. In Cluster 2, our original input dataset without delaying processes of population flux data gets better prediction performance. The optimal population flux delayed time for Cluster 3 is two months based on XGBoost prediction model.

## Discussion

In this paper, we used two machine learning methods, Random Forest and XGBoost, to perform our analysis and prediction on HFMD incidence. There have been a large number of studies focusing on machine learning methods to analyze different infectious diseases, and to perform prediction about the incidence of diseases, such as dengue [30, 33–35], polio [36], human brucellosis [37], malaria [38], and COVID-19 [39, 40]. It is also applied for the prediction of HFMD from meteorological factors in a single province in China [41]. Several studies using machine learning methods compared the performance of different prediction methods [30, 34, 37]. A study on human brucellosis in mainland China stated that XGBoost model is more suitable for prediction cases of human brucellosis in mainland China than ARIMA model [37]. Our study did not involve ARIMA model, but we compared the prediction performance of XGBoost model and Random Forest model. We found that XGBoost model is more suitable for predicting the incidence of HFMD in mainland China than Random Forest model.

Our finding that temperature is the most important meteorological factor affecting the transmission of HFMD is consistent with the results of those studies that only considered meteorological factors [16–19, 42–44]. However, we also found that social factors, including population flux and population density, also affect the incidence of HFMD. A study using regression model found that meteorological factors and population density are potential determinants of the HFMD incidence in most areas in China, but population flux was not considered in it [45]. Population flux effects on disease incidence should not be ignored when we study incidence patterns. Population flux was also found to have effects on transmission rate of measles and rubella [13, 22]. Meanwhile, an increase in transmission rate happened during a forty-day period of Chinese Spring Festival Travel Rush and assumed to be related to the high level of population flux [3]. Moreover, our results showed that population flux has different delayed effect in affecting HFMD incidence in different regions. Most time delayed effect studies on HFMD is about meteorological factors [16, 17]. The delayed effect of population mobility on transmission rates of rubella, HFMD, and COVID-19 were found by using transmission models [3, 13, 14].

We noticed that a study excluded average pressure factor when analyzing the influential factors on the incidence of infectious diseases [38]. In our initial research, we have taken average pressure factor along with other meteorological factors into account. Our initial model showed that average pressure has almost the same importance as average temperature. However, we found that, the correlation coefficients of average pressure and average temperature are -0.9389 and -0.9386 respectively through two correlation tests, Spearman and Pearson. It indicates that average pressure and average temperature have a strong negative correlation. In order to eliminate the confounding effect between factors, we excluded average pressure factor in this study.

There are some limitations in our study. We did not consider the effect of population flux within a province because of the limitation of our data. It requires finer scale epidemic data such as incidence at county level, which is not the case in this study. The capability of our prediction model needs to be improved. The main reason that affects the performance of our model is the small amount of the data. From Table 3, we can find that the MSE value of Cluster 3 is higher than the MSE value at the country level. It requires us to optimize our prediction model while expanding our data. In our future studies, we should also consider whether factors in other fields, such as control measures or economic factors, will affect HFMD incidence. We will explore more machine learning algorithms, such as the LSTM in the recurrent neural network, to establish a more suitable model for predicting the incidence of HFMD in mainland China.

The purpose of our study is to provide references for individuals, hospitals and clinics to prevent and control HFMD, and to help them develop preventive measures and minimize health risks.

## Conclusion

Average temperature and population density are the two most significant factors affecting HFMD incidence. Population flux has different delayed effect in affecting HFMD incidence in different regions of mainland China. XGBoost model is more suitable for predicting the incidence of HFMD in mainland China.
